# Mean Auricular Index of the External Ear in Medical Students of a Medical College in Nepal

**DOI:** 10.31729/jnma.4636

**Published:** 2019-10-31

**Authors:** Niraj Pandey, Sudikshya KC, Chandan Sintakala

**Affiliations:** 1 Department of Anatomy, Lumbini Medical College and Teaching Hospital, Palpa, Nepal

**Keywords:** *ear lobule*, *external ear*, *human auricle*, *mean auricular index*

## Abstract

**Introduction:**

The human ear is divided into external, middle and internal ear. The auricle helps in the clinical diagnosis of congenital anomalies and syndromes. The aim of this study is to determine the mean auricular index from the right and left ears, mean measurements of left and right ear and sex-related dimensions of the ear.

**Methods:**

A descriptive cross-sectional study was conducted in medical students studying at a tertiary care hospital during the period of 1st April 2019 to 20^th^ May 2019 after ethical clearance from the institutional review committee. Simple random sampling was done. Data were collected, entered in Statistical Package for Social Sciences version 20 and point estimate at 95% Confidence Interval was calculated along with frequency and proportion for binary data.

**Results:**

Out of total measurements done, we found the mean auricular index was found to be for right ear 51.75±4.51mm and for left ear 54.09±4.96mm at 95% Confidence Interval (51.75-52.37) and (53.43-54.77) respectively. The mean ear length, mean ear breadth, mean lobular length and mean lobular breadth were 57.62±5.03mm, 29.72±2.79mm, 19.32±2.60mm and 20.89±3.06mm on the right side and 57.89±4.89mm, 31.21±2.95mm, 19.38±2.55mm and 21.25±2.81mm on the left side respectively.

**Conclusions:**

The mean auricular index of the external ear of medical students was within the normal range as compared to the other studies conducted.

## INTRODUCTION

The auricle collects sound waves and conducts them along the external acoustic meatus inwards towards the eardrum, the tympanic membrane. Width dimensions mature between the ages of 5 and 11 years; length dimensions mature between 12 and 16 years.^1^ Variations in-ear proportions during growth, aging, and according to sex were reported.

The auricle helps in the clinical diagnosis of congenital anomalies and syndromes. For a physician, a deformation in auricular shape and size can point toward a possible anomaly in the patient. Trisomy 13 and 18 syndromes are reported to have low-set and deformed auricles, whereas Down’s syndrome patients have smaller auricles than normal.^2^ The auricle is one of the five primary features of the human face and is particularly influential in determining its appearance.^3^

The aim of this study is to determine the mean auricular index from the right and left ears, mean measurements of left and right ear and sex-related dimensions of the ear.

## METHODS

A descriptive cross-sectional study was conducted at the Department of Anatomy, Lumbini Medical College and Teaching Hospital, Pravas, Palpa, Nepal from 1st April 2019 to 20th May 2019. Ethical clearance was taken from the institutional review committee of Lumbini Medical College and Teaching Hospital. The study population was MBBS students presently studying at Lumbini Medical College and Teaching Hospital, aged between 18 and 24 years were selected as the subjects for the present study with no evidence of congenital ear anomalies or previous ear surgeries. The inclusion criteria for participants in the study is medical students studying in the college. Exclusion criteria for participants in the study were students with previous ear surgeries, ear deformities, and presence of any ear diseases.

Simple random sampling technique was used and the sample size was calculated using the following formula,

n = Z^2^ × S.D.^2^/e^2^

   = 1.96 × 1.96 × (0.71)^2^/(0.1)^2^

   = 188.16

Where,

n = sample size
S.D. = standard deviation (0.71)
e = margin of error (10%)
Z = 1.96 at 95% CI

The total sample size for the study was taken as 200 participants. A fixed time from 11 am to 4 pm was selected for the physical measurement to eliminate the discrepancies due to diurnal variation. The measurements were taken with the help of a vernier caliper. Students were informed about the study design, its benefits, and the privacy of the data collected. Bilateral sizes of auricles were measured. Standardized measurements of the ear pinna were taken according to the landmarked points defined by De Carlo et al. and the methodology was adopted from McKinney et al. and Brucker et al. the parameters measured were total ear height (TEH), ear width (EW), lobular height (LH), and lobular width (LW) for each subject’s right and left ears, when the head was in the Frankfort horizontal plane, shows the measurement of ear reference points used for anthropometric measurements.

The total ear length was measured as the distance from the most inferior projection of the ear lobule to the most superior projection of the helix. The ear breadth is measured as the distance between the most anterior and posterior points of the ear. The lobular length was taken as the distance from the most inferior end of the lobule to the base of the tragal notch. The lobular width was measured as the transverse or horizontal width of the lobule. Additionally, the indices defining the proportions of the ear such as ear index were calculated. Auricular index (AI): Width of auricle/length of the auricle×100

Selection and information bias has been minimized as possible. Data were collected, entered in SPSS version 20 and point estimate at 95% Confidence Interval was calculated along with frequency and proportion for binary data.

## RESULTS

In a total of 200 medical students, the mean auricular index was found to be for right ear 51.75±4.51mm and for left ear 54.0996±4.95682mm at 95% C.I. (51.75-52.37) and (53.426-54.766) respectively (Table 1).

**Table 1:** Auricular index of right and left ear (n=200).

Auricular Index	Minimum (mm)	Maximum (mm)	Mean±SD (mm)
Right ear	38.90	65.63	51.7585±4.51087
Left ear	43.46	70.19	54.0996±4.95682

The mean ear length, mean ear breadth, mean lobular length and lobular breadth were 57.62±5.03mm, 29.72±2.79mm, 19.32±2.60mm and 20.89±3.06mm on the right side and 57.89±4.89mm, 31.21±2.95mm, 19.38±2.55mm and 21.25±2.81mm on the left side respectively (Table 2).

**Table 2:** Measured ear variables (n=200).

Variables	Minimum (mm)	Maximum (mm)	Mean±SD (mm)
age of students	18	24	19.79±.938
length of the right ear	47.31	70.46	57.6249±5.03763
length of the left ear	44.81	71.41	57.8898±4.88770
breadth of the right ear	22.84	41.45	29.7297±2.79036
breadth of the left ear	23.19	39.66	31.2158±2.94923
length of the right lobule	13.77	38.91	19.3247±2.60539
length of the left lobule	14.33	26.61	19.3818±2.55432
breadth of right ear lobule	14.69	28.46	20.8864±3.06475
breadth of left ear lobule	14.12	30.38	21.2518±2.81746

The variations of different ear measurements in different gender have been measured and tabulated (Table 3).

**Table 3:** Variations of variables in different gender.

Variables	Gender	Sample (n)	Mean±SD	Std. Error of Mean
length of right ear	Male	110	59.3025±4.71758	.44980
	Female	90	55.5747±4.66453	.49168
length of the left ear	Male	no	59.6906±4.31409	.41133
	Female	90	55.6887±4.65991	.49120
breadth of the right ear	Male	110	30.6278±2.74479	.26171
	Female	90	28.6319±2.44271	.25748
breadth of the left ear	Male	110	31.7757±2.82227	.26909
	Female	90	30.5313±2.97184	.31326
length of the right lobule	Male	110	19.2020±3.06470	.29221
	Female	90	19.4746± 1.90617	.20093
length of the left lobule	Male	110	18.9453±2.65152	.25281
	Female	90	19.9154±2.33553	.24619
breadth of right ear	Male	110	20.9535±3.27722	.31247
lobule	Female	90	20.8043±2.79921	.29506
breadth of left ear lobule	Male	110	21.5628±2.77233	.26433
	Female	90	20.8716±2.84084	.29945

## DISCUSSION

The auricular length and width can be useful in diagnosing syndromes including microtia or craniofacial syndromes that may present with disproportionately wide or narrow ears and the total ear length is important in the evaluation of congenital anomalies like Down syndrome.^4,5^ The ear reaches its mature height at 13 years in males and at 12 years in females.^6^ the study by McKineey et al. addressed specifically the treatment of the ear and earlobe in esthetic surgery and obtained data from 100 normal volunteers and found a mean ear height of 6.50cm and a mean LH of 1.80cm, with no significant correlation between the earlobe height and aging.^7^

In a study consisting of North American Whites, it was observed that the total height of the left ear was 62.4mm in men and 58.5mm in women,^8^ and that the same measurement was 70.1 mm in Japanese people^9^ but in our study we found the total ear length, total ear width, lobular length, and lobular breadth were 57.62mm, 29.72mm, 19.32mm and 20.89mm on the right side and 57.89mm, 31.21mm, 19.38mm and 21.2 5mm on the left side respectfully. The present study focused on anthropometric measurements of TEL, EW, LL, and LW of both sides. In a similar study in Maiduguri, Northern Nigeria by Ekanem et al. the total mean values of TEL, LL, and LW when the data were added were lower than in the present study.^10^

The knowledge of morphometric parameters of different landmarks on the face in relation to different age groups and gender has become essential in the modern era for accurate plastic reconstruction and forensic purposes.^10,11^ The knowledge of normal ear dimensions may be useful as a guideline for the plastic surgeon, forensic purposes and for the industrial manufacturing hearing instruments. Lobule length and width in our study are higher in females as compared to males. This fact is supported by another study done on the Urhobo people of South Nigeria.^12^ In contrast to this, Wang et al. suggested that lobule dimensions are not significantly different in two genders.^13^ When the right and left sides were compared, the recent study showed no statistically significant asymmetry in the ear. The study done by Bozkir et al. found the morphometric measurements from the right and left ears also observed similar symmetry among them.^14^ The study was done by Sforza et al. in Italian Caucasians had determined the age and sex-related changes in the normal and healthy human ear observed that with age, all measurements were larger in men than in women of corresponding age.^15^ The study done by Adamson et al. found that after 20 years of age, the changes in the appearance of pinna occur due to the rearrangement of elastic fibers.^16^ The changes in the size of lobule are mainly seen after 45 years of age due to gravitational forces and also secondary to wearing of ornaments by females (Barut and Aktunc, 2006).^17^ In the study done by Kalra et al. they found the Ear Index of right and left ear as 50.23±3.97mm, and 50.30±4.35mm respectfully and Lobule Index of right and left ear as 118.25±16.95mm, and 117.11±15.87mm respectfully^18^ whereas in our study we found the ear index and lobule index of right and left ear as 51.76±4.51mm, 54.09±4.95mm, 108.88±15.50mm and 110.9296±17.01mm respect fully. When we compare our study with those of others, we find that there is a difference in the values of ear measurements, and these discrepancies could be a result of factors such as race, genetic variables, individual constitution, environment, age, and human error.

## CONCLUSIONS

Mean auricular index of the external ear of medical students was within the normal range as compared to the other studies conducted. This study provides mean values of different measurements of right and left ears of both the sexes of age group of 18-24 years in Nepalese population which is helpful for plastic surgeons, designing of hearing aids and instruments and for forensic scientists.

## References

[1] Standring S (2008). Gray’s Anatomy. 40th ed..

[2] Vogel FG, Motulsky AG (1982). Human genetics: Problem and approaches.

[3] Purkait R, Singh P (2007). Anthropometry of the normal human auricle: A study of adult Indian men. Aesthetic Plast Surg..

[4] Farkas LG, Posnick JC, Hreczko TM (1992). Anthropometric growth study of the ear. Cleft Palate Craniofac J..

[5] Chou CT, Tseng YC, Tsai FJ, Lin CC, Liu CS, Peng CT, et al (2002). Measurement of ear length in neonates, infants and preschool children in Taiwan. Acta Paediatr Taiwan..

[6] Ito I, Imada M, Ikeda M, Sueno K, Arikuni T, Kida A (2001). A morphological study of age changes in adult human auricular cartilage with special emphasis on elastic fibers. Laryngoscope.

[7] McKinney P, Giese S, Placik O (1993). Management of the ear in rhytidectomy. Plast Reconstr Surg..

[8] Brucker MJ, Patel J, Sullivan PK (2003). A morphometric study of the external ear: age and sex-related differences. Plast Reconstr Surg..

[9] Asai Y, Yoshimura M, Nago N, Yamada T (1996). Why do old men have big ears? Correlation of ear length with age in Japan. BMJ.

[10] Ekanem AU, Garba SH, Musa TS, Dare ND (2010). Anthropometric study of the pinna (Auricle) among adult Nigerians resident in Maiduguri metropolis. J Med Sci..

[11] Sharma A, Sidhu NK, Sharma MK, Kapoor K, Singh B (2007). Morphometric study of ear lobule in Northwest Indian male subjects. Anat Sci Int..

[12] Eboh D (2013). Morphological changes of the human pinna in relation to age and gender of Urhobo people in Southern Nigeria. J Exp Clin Anat..

[13] Wang B, Dong Y, Zhao Y, Shizhu BS, Wu G (2011). Computed tomography measurement of the auricle in Han population of North China. J Plast Reconstr Aesthet Surg..

[14] Bozkir MG, Karakas P, Yavuz M, Dere F (2006). Morphometry of the external ear in our adult population. Aesthet Plast Surg..

[15] Sforza C, Dellavia C, Tartaglia GM, Ferrario VF (2005). Morphometry of the ear in Down’s syndrome subjects: A three-dimensional computerized assessment. Int J Oral Maxillofac Surg..

[16] Adamson JE, Hortox CE, Crawford HH (1965). The growth pattern of the external ear. Plast Reconstr Surg..

[17] Barut C, Aktunc E (2006). Anthropometric measurements of the external ear in a group of Turkish primary school students. Aesthet Plast Surg..

[18] Kalra D, Kalra A, Goel S (2015). Anthropometric measurements of external ear: An in vivo study. Int J Enhanced Res Med Dent Care.

